# Effect of furosemide on body composition and urinary proteins that mediate tubular sodium and sodium transport—A randomized controlled trial

**DOI:** 10.14814/phy2.14653

**Published:** 2020-12-23

**Authors:** Frank Holden Mose, Anna Ewa Oczachowska‐Kulik, Robert Andrew Fenton, Jesper Nørgaard Bech

**Affiliations:** ^1^ University Clinic in Nephrology and Hypertension Department of Medicine University of Aarhus and Gødstrup Hospital Holstebro Denmark; ^2^ Department of Biomedicine Aarhus University Aarhus Denmark

**Keywords:** body composition, furosemide, sodium‐potassium‐chloride cotransporter

## Abstract

**Background:**

Furosemide inhibits the sodium potassium chloride cotransporter (NKCC2) in the thick ascending limb of the loop of Henle and increases urinary water and sodium excretion. This study investigates the effect of furosemide on body composition estimated with multifrequency bioimpedance spectroscopy (BIS) technique and urinary proteins from NKCC2.

**Methods:**

This study is a randomized, placebo‐controlled, crossover study where healthy subjects received either placebo or 40 mg furosemide on two separate occasions, where body composition with BIS, renal function, proteins from tubular proteins that mediate sodium and water transport, and plasma concentrations of vasoactive hormones were measured before and after intervention.

**Results:**

We observed an expected increased diuresis with a subsequent reduction in bodyweight of (−1.51 ± 0.36 kg, *p* < .001) and extracellular water (ECW; −1.14 ± 0.23 L, *p* < .001) after furosemide. We found a positive correlation between the decrease in ECW and a decrease in bodyweight and a negative correlation between the decrease in ECW and the increase in urinary output. Intracellular water (ICW) increased (0.47 ± 0.28 L, *p* < .001). Urinary excretion of NKCC2 increased after furosemide and the increase in NKCC2 correlated with an increase in urine output and a decrease in ECW.

**Conclusion:**

We found BIS can detect acute changes in body water content but the method may be limited to estimation of ECW. BIS demonstrated that furosemide increases ICW which might be explained by an extracellular sodium loss. Finally, urinary proteins from NKCC2 increases after furosemide with a good correlation with diuresis end the decrease in ECW.

## Graphical abstract

We found that BIS can detect acute changes in body water content but the method may be limited to estimation of ECW. BIS demonstrated that furosemide increases ICW which might be explained by an extracellular sodium loss. And finally urinary proteins from NKCC2 increases after furosemide with a good correlation with diuresis end the decrease in ECW.

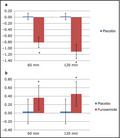

## 1 BACKGROUND

The kidneys regulate fluid and sodium homeostasis which becomes evident in renal failure where fluid retention is often present (Yerram et al., 2010). The mechanisms for fluid retention are not completely described but includes decreased number of nephrons, abnormal activity of tubular cells that regulate water and sodium excretion, abnormal function of vasoactive hormones that regulate fluid and sodium homeostasis including the renin‐angiotensin‐system and natriuretic peptides and change in central blood volume and blood pressure that lead to change in renal perfusion, activity in vasoactive hormones and sympathetic nervous activity (Raina et al., 2018; Zoccali et al., 2017). Intuitively the kidney must play a central role in the regulation of the different fluid compartments in the body such as intracellular and extracellular volume but this is not well understood (Matthie, 2008; Wabel et al., 2009). Methods using multifrequency bioimpedance spectroscopy (BIS) technique may help to improve our understanding of body composition, fluid regulation, and treatment of volume overload in different stages of renal dysfunction (Arroyo et al., 2015; Ersoy Dursun et al., 2019; Hur et al., 2013; Lukaski et al., 2019; Onofriescu et al., 2014). The activity of tubular proteins that mediate sodium transport is difficult to measure directly but surrogate markers such as protein fragments from transporters have previously been used (Al Therwani et al., 2017; Graffe et al., 2012; Jensen et al., 2014; Pedersen et al., 2001). The effect of furosemide on urinary excretion of proteins from the furosemide sensitive sodium potassium chloride cotransporter (NKCC2) has to our knowledge not been investigated previously (Huang et al., 2016).

We therefore hypothesized that furosemide treatment increases urine flow and causes a reduction in bodyweight associated with reductions in ECW and ICW measured with BIS. In addition, the effect of furosemide will change u‐NKCC2 reflecting u‐NKCC2 activity. Finally, the changes in u‐NKCC2 are associated with changes in fluid distribution in the body. We investigated these hypotheses in a study designed as a randomized, placebo‐controlled, crossover study where subjects received either placebo or furosemide on two separate occasions, where body composition, renal function, proteins that mediate tubular sodium and water transport, and plasma concentrations of vasoactive hormones were measured.

## 2 METHODS

### 2.1 Design

**FIGURE 1:**
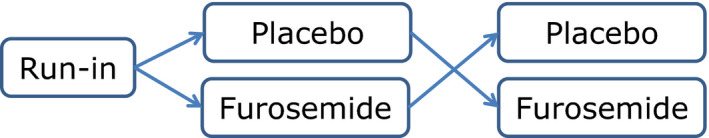
Study design

The study was a randomized, single‐blinded, placebo‐controlled, crossover trial (Figure 1). After inclusion subjects were allocated to treatment via computer‐generated randomization and received furosemide or 5% glucose (placebo) on examination days in a random order. Examinations were separated by a washout period of at least 2 weeks.

Furosemide (Furix, 4 ml of 10 mg/ml, Nycomed Danmark) and isotonic glucose (4 ml 50 g glucosemonohydrate/l, Baxter) were identical in appearance to the study subjects. Furosemide was given at a dose of 40 mg (4 ml) intravenously. Glucose was chosen as placebo to minimize sodium intake.

### 2.2 Effect variables

Extracellular water (ECW) was chosen as the main effect variable. Other effect variables were intracellular water (ICW), ECW/ICW, bodyweight, GFR, plasma sodium (p‐Na), serum osmolality, FE_Na_ (fractional excretion of sodium), free water clearance (C_H2O_), urinary excretions of aquaporin‐2 (u‐AQP2), epithelial sodium channels (u‐ENaC_γ_), sodium chloride cotransporter (u‐NCC) and sodium potassium chloride cotransporter (u‐NKCC2), urinary osmolality, plasma concentration of vasopressin (p‐AVP), renin (PRC), angiotensin II (p‐AngII) and aldosterone (p‐Aldo), brachial systolic and diastolic blood pressure (DBP, SBP), and heart rate (HR).

### 2.3 Recruitment

Subjects were consecutively recruited by advertisements in local newspapers in the area of Holstebro, Denmark. Written and oral information that included safety concerns of study medication was given, following a written consent. After the written consent was obtained the screening examination was performed. A clinical history was taken and examination was performed, blood was drawn and urine samples were collected. ECG was performed to ensure that the subject fulfilled the inclusion criteria and did not meet the exclusion criteria. Screening examination included physical examination, medical history, clinical biochemistry, urine albumin analysis ECG, and ambulatory BP measurement.

### 2.4 Subjects

Inclusion criteria: Healthy women and men, BMI 18.5–30.0 kg/m^2^, age 18–45 years, fertile women must use safe anticonception. Exclusion criteria: Clinical signs of or history with diseases in the central nervous system, thyroid gland, heart and lungs, liver or kidneys, malignancies, diabetes mellitus, ambulatory blood pressure >130 mmHg systolic and/or >80 mmHg diastolic, clinical important deviations in screening urine or blood samples, medical or alcohol abuse, smoking, nursing or pregnancy, allergy or intolerance towards furosemide, unwillingness to participate. Withdrawal criteria: Noncompliance or development of exclusion criteria.

### 2.5 Number of subjects

With a significance level of 5% and a power of 80% a total of 22 subjects were needed to detect a 1.25 L difference in ECW (*SD* 2 L). Because incomplete voiding during examination days was expected in some subjects, it was estimated that 24 subjects should finish the trial.

### 2.6 Experimental procedure

Prior to examinations subjects received a 4‐day standard diet, as previously described (Jensen et al., 2013, 2014; Matthesen et al., 2013; Mose et al., 2015). The diet comprised three minor meals and three main meals. The complete diet contained 11,000 kJ/day and was composed of 55% carbohydrates, 30% fat, and 15% protein. The total sodium content was 150 mmol per day. Subjects were instructed to eat variedly from the diet until satiated. Daily fluid intake was recommended to 2.75 L (250 ml per 1,000 kJ). No consumption of alcohol was allowed. Up to two cups of coffee or tea pr. day were allowed.

Twenty‐four‐hour urine collection was performed before each examination. Twenty‐four‐hour urine was analyzed for sodium, creatinine, albumin, AQP2, ENaC_γ_, NCC, and NKCC2. After an overnight fast, subjects arrived for examination at 8 a.m. Two indwelling catheters for blood sampling and administration of ^51^Cr‐EDTA and furosemide or glucose (placebo) were placed in cubital veins, one in each arm. Every 30 min after arrival, participants received an oral water load of 175 ml of tap water. Subjects were kept in a supine position in a quiet, temperature‐controlled room (22°C–25°C). Standing or sitting was only permitted during voiding. At 11 a.m. injection furosemide or glucose was given according to randomization.

Blood and urine samples were collected every 30 min from 9:30 a.m. to 2.30 p.m. Urine collections were analyzed for ^51^Cr‐EDTA, sodium, creatinine, AQP2, ENaC_γ_, NCC, and NKCC2. The first three clearance periods from 9:30 a.m. to 11 a.m. were used as the baseline period. The baseline period was followed by clearance periods as described above.

Blood samples were drawn at 11 a.m. (baseline) just prior to infusion of study medication and again 1 and 2 hr after infusion of study medication for determination of p‐AVP, PRC, p‐AngII, and p‐Aldo.

### 2.7 Blood pressure measurements

Office BP measured during examination was recorded by the semiautomatic, oscillometric device, Omron 705IT (Omron Matsusaka CO. Ltd.). Bioimpedance spectroscopy.

Bioimpedance spectroscopy (BIS) was measured using Body Composition Monitor (BCM, Fresenius Medical Care) and was used according to the manufacturer's instructions. Bioimpedance measurements performed at a spectrum of 50 frequencies between 5 and 1,000 kHz allow to differentiate between extra‐ and intracellular fluid, as low electronic currents only flow through extracellular water because they cannot pass cell membranes (Moissl et al., 2006). Parameters of volume status and body composition are calculated by the BCM using two physiological models: The body volume model is used to calculate ECW, ICW, and total body water (TBW) and the body composition model differentiates normally hydrated fat mass, normally hydrated lean mass and a remaining proportion of water, and lays the foundation to calculate parameters of adipose tissue, lean tissue and the so‐called overhydration (OH; Chamney et al., 2007). OH is mainly part of extracellular fluid and reference values for OH lie between −1 and +1 L.

### 2.8 Biochemical analyses

Urine samples were kept frozen at −20°C until assayed. U‐AQP2, u‐ENaC_γ_, and U‐NKCC2 were measured by radioimmunoassay as previously described (Al Therwani et al., 2017; Graffe et al., 2012; Jensen et al., 2014; Pedersen et al., 2001). Antibodies were raised in rabbits to synthetic peptides for AQP2, ENaC_γ_, and NKCC2.

Urine samples for measurement of NCC were thawed and centrifuged at 2,200 *g* for 10 min before storage. A sample volume—standardized to osmolality—was freeze‐dried and kept at −20°C until analysis. For analysis, the freeze‐dried samples were suspended in 200 µl albumin buffer (phosphate 40 mM, albumin 2 g/L) and 100 µl assay buffer. Assay buffer contained 40 mM phosphate, albumin 2 g/L, 0.36% EDTA, and 1 ‰ Triton‐X‐100. 50 µl of antibody was added to each tube and incubated for 24 hr at 4°C. 50 µl of ^125^I‐NCC was added and incubated for further 24 hr. 100 µl of bovine gamma globulin and 2 ml of polyethylene glycol were added. After 1 hr, the tubes were centrifuged at 4,100 *g* for 20 min. at 4°C. The supernatant was discarded and the precipitate was counted in a gamma counter. A standard curve was constructed (i.e., 9 points increasing from 0 pg/tube to 4,000 pg/tube) to read of the unknown amounts of NCC in urine extracts.

For six consecutive standard curves, the zero standard was 81.3 ± 1.4% binding. For increasing amounts of NCC‐standard, the binding inhibition was 79.6 ± 1.3% (31 pg/tube), 77.7 ± 1.3% (62.5 pg/tube), 73.0 ± 1,8% (125 pg/tube), 64.1 ± 1,6% (250 pg/tube), 44.9 ± 2.1% (500 pg/tube), 26.9 ± 1.0% (1,000 pg/tube), 17.5 ± 0.7% (2,000 pg/tube), and 12.1 ± 0.3% (4,000 pg/tube). The minimal detection limit was 62.5 pg/tube (zero binding −2 SD). Average nonspecific binding was 5.7 ± 0.6%. The ID 50 (concentration of standard needed for 50% binding inhibition) was 575 ± 34 pg/tube. The intraassay coefficient of variation was 8.2% (*n* = 40, 4 assays) and interassay coefficient of variation was 11.1% (*n* = 36, 9 assays). Iodination was obtained using chloramine T with 40 µ antigen and 37 MBq I^125^. I^125^‐NCC was separated on a G25 Sephadex column after the process was terminated using 20% human albumin. NCC was obtained from Genscript Biotech (Netherlands).

The NCC antibody was produced and specificity was secured. A 18‐amino acid peptide, CRRDCPWKISDEEITKNR (the NH2 terminal cysteine added for conjugation) corresponding to amino acids 943–959 of human NCC (accession# AAC50355.1) was produced by standard solid‐phase techniques and conjugated to keyhole limpet hemocyanin (KLH) via covalent linkage to the NH2‐terminal cysteine (Genscript USA). The antibody was affinity purified (termed #8285) from terminal bleed serum using the immunizing peptide as described previously (Fenton et al., 2007). The antibody #8285 titer was determined to be >1:512,000 using ELISA and NCC peptide‐conjugated plates. Antibody #8285 specificity was determined by: (a) western blotting of MDCK cells expressing human NCC, where a strong signal was only observed in transfected cells (Rosenbaek et al., 2017); (b) western blotting of human whole kidney, cortex or medulla tissue, showing a strong band of the characteristic molecular mass of NCC only in cortical samples (c) triple immunohistochemical labeling (as previously described) of mouse kidney using markers of late DCT (CalbindinD28) and connecting tubule/collecting duct (Aquaporin‐2; Pedersen et al., 2010); (d) immunohistochemical labeling of tubules morphologically similar to the distal convoluted tubule in human kidney sections.

Blood samples collected for measurements of vasoactive hormones were centrifuged and plasma was separated, and kept frozen until assayed as previously described (Al Therwani et al., 2014). AVP and Ang II were extracted from plasma and then determined by radioimmunoassay (Al Therwani et al., 2014; Pedersen et al., 1984, 1993). PRC was determined by immunoradiometric assay as previously described (Al Therwani et al., 2014). Aldo was determined by radioimmunoassay as previously described (Mose et al., 2014).

Glomerular filtration rate (GFR) was determined with constant infusion clearance technique with ^51^Cr‐EDTA as a reference substance (Jensen et al., 2014; Mose et al., 2015). Urine and plasma concentration of creatinine, sodium, and albumin were measured by routine methods at the Department of Clinical Biochemistry.

### 2.9 Calculations

In 24‐hr urine collections, GFR was estimated by creatinine clearance. Fractional excretions of sodium (FE_Na_) was calculated with the formula: FE_Na_ = (U_Na_ * V/C_Na_)/GFR, where U_Na_ and C_Na_ are urine and plasma concentrations of Na^+^ and V is urine flow in ml/min. Free water clearance (C_H2O_) was calculated using the formula: C_H2O_ = UO − C_osm_, where UO is urinary output and C_osm_ is osmolar clearance.

Urine concentration of variables is adjusted for urinary flow resulting in an excretion rate and for creatinine excretion giving an approximate adjustment for glomerular filtration.

### 2.10 Statistics

Data are presented as medians with 25% and 75% percentiles in brackets, if normality was not present and as means ± standard deviations (*SD*), if data showed normality. A paired comparison within and between groups was performed with paired *t* test or Wilcoxon signed‐rank test. A general linear model for repeated measures (GLM) was performed to test the difference in responses to furosemide during the experimental procedure. If normality was not present, data were logarithmic transformed before GLM. Friedman's test was used to test if deviations within the treatment of vasoactive hormones occurred during the experimental procedure. Correlations were performed with Pearson correlation. Statistical significance was defined as *p* < .05. Statistical analyses were performed using PASW version 20.0.0 (SPSS Inc.).

### 2.11 Ethics

The study was approved by the Danish Health and Medicines Authority (EudraCT number: 2012‐003815‐71) and the Regional Committee on Biomedical Research Ethics (case number:1‐16‐02‐540‐14). It was carried out in accordance with the Declaration of Helsinki and was monitored by the Good Clinical Practice Unit from Aarhus and Aalborg Universities. A signed informed consent form was obtained from each patient.

## 3 RESULTS

### 3.1 Demographics

Forty subjects were screened for participation and included in the trial. 19 subjects were excluded due to medication use (1), low potassium (1), anemia (1), hypertension (1), elevated alaninaminotransferase (1), smoking (1), heart murmurs (2), abnormal ECG (1), no possible cubital intra‐venous access and withdrawal of consent (8). Thus, 21 healthy subjects were included and completed the trial. Twenty‐one subjects (13 females, 8 males), had a mean BMI 24.1 ± 2.5 kg/m^2^, age 26 ± 5 years, ambulatory BP 118/71 ± 8/6 mmHg, p‐creatinine 73 ± 10 μmol/L, urine albumin 6 (1;9) mg/L, p‐hemoglobin 8.6 ± 0.7 mmol/L.

### 3.2 Bioimpedance spectroscopy and bodyweight

**FIGURE 2:**
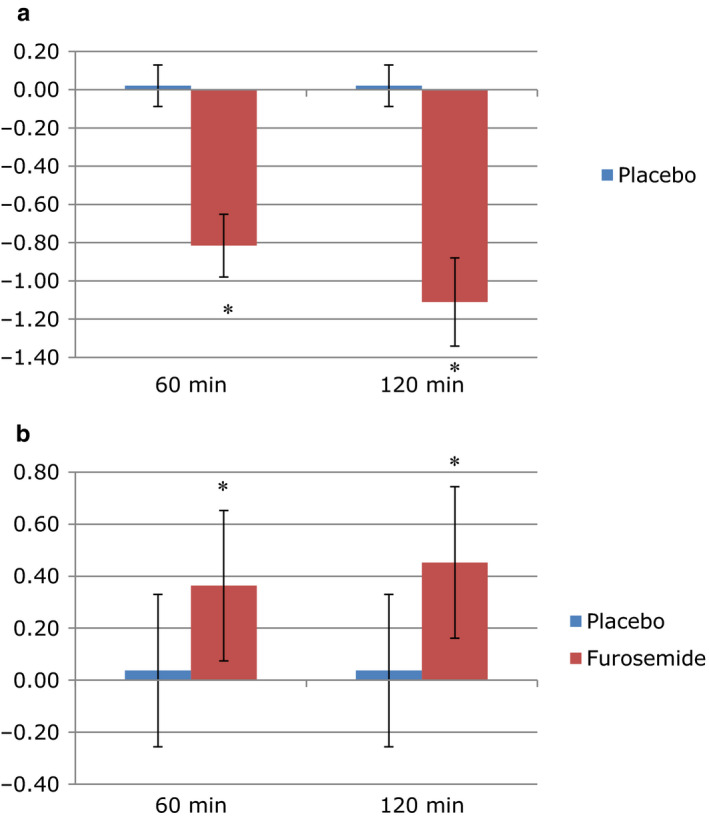
Change in ECW (a) and ICW (b) 60 and 120 min after furosemide and placebo infusion. Statistically significant difference from baseline: * = *p* < .05

At baseline, ECW, ICW, OH, ECW/ICW, and bodyweight were similar between treatments (Table 1). OH was negative at baseline indicating small dehydration, which was attenuated after furosemide. Furosemide reduced bodyweight (−1.51 ± 0.36 kg, *p* < .001). The change in ECW and ICW from baseline is shown in Figure 2. The ECW/ICW ratio was reduced after furosemide.

**TABLE 1:** Effect of furosemide on bioimpedance spectroscopy (BIS) and plasma sodium and osmolality

	Baseline	1 hr post intervention (60 min)	2 hr post intervention (60 min)	*p*‐value (difference in response)
ECW (L)				
Placebo	15.9 ± 3.4	15.9 ± 3.4	15.9 ± 3.4	<.001
Furosemide	15.7 ± 3.7	14.9 ± 3.5*	14.5 ± 3.5*	
ICW (L)				
Placebo	22.5 ± 6.2	22.6 ± 6.1	22.6 ± 6.0	<.001
Furosemide	22.5 ± 6.4	22.9 ± 6.4*	23.0 ± 6.6*	
ECW/ICW				
Placebo	0.71 ± 0.05	0.71 ± 0.04	0.71 ± 0.04	<.001
Furosemide	0.71 ± 0.04	0.66 ± 0.04*	0.64 ± 0.04*	
Overhydration (L)				
Placebo	−0.7 ± 0.8	−0.7 ± 0.8	−0.8 ± 0.9	<.001
Furosemide	−0.9 ± 0.9	−1.8 ± 0.8	−2.1 ± 0.7	
Bodyweight (kg)				
Placebo	70.7 ± 10.8	70.6 ± 10.9*	70.5 ± 10.9*	<.001
Furosemide	70.6 ± 10.4	69.4 ± 10.6*	69.1 ± 10.5*	
P‐Sodium (mmol/l)				
Placebo	139 ± 1	138 ± 1*	138 ± 1*	.205
Furosemide	138 ± 1	138 ± 1	137 ± 1*	
S‐Osmolality (mmol/kg)				
Placebo	285 ± 4	284 ± 4*	282 ± 4*	.423
Furosemide	285 ± 5	284 ± 4	281 ± 5*	

Note

Extracellular volume (ECW), intracellular volume (ICW), bodyweight, p‐sodium, and s‐osmolality were measured before furosemide or placebo infusion and repeated 1 and 2 hr after infusion. Data are shown as means ± *SD*. *p*‐value represents the probability of difference in response to furosemide (response from baseline to 1 hr after injection) between treatments. Students *t* test was used to test the difference in response to injection between treatments and to test statistically significant difference from baseline, *p < .05.

### 3.3 Plasma sodium and serum osmolality

P‐sodium and u‐osmolality decreased after both placebo and furosemide and to a similar extent (Table 1).

### 3.4 GFR and tubular function during baseline conditions

The volume and composition of 24‐hr urinary collection made prior to the two examinations were not significantly different between treatments (Table 2). Similarly, at baseline during examinations, no difference in evaluated parameters was found (Table 3), GFR, urine output, C_H2O_, FE_JNa_, U‐AQP‐2, u‐ENaC_γ,_ u‐NCC, and u‐NKCC2 were similar between treatment arms (Tables 3 and 4).

**TABLE 2:** 24‐hr urine collection prior to two examinations

	Placebo	Furosemide	*p*‐value
Urine output (ml/minute)	1.68 ± 0.61	1.68 ± 0.47	1.000
C_H2O_ (ml/minute)	−0.21 ± 0,54	−0.30 ± 0.57	.578
U‐creatinine (mmol/24 hr)	14.7 ± 4.4	14.8 ± 3.6	.950
Creatinine clearance (mmol/mL pr. m^2^)	137 ± 29	139 ± 25	.772
U‐Na (mmol/24 hr)	117 ± 49	111 ± 27	.570
FE_Na_ (%)	20.1 ± 13	23 ± 18	.387
UAER (mg/24 hr)	5 (4;12)	7 (5;12)	.938
U‐AQP−2/min (ng/minute)	1.12 ± 0.32	1.12 ± 0.29	.187
U‐AQP−2/creatinine (ng/mmol)	111 ± 15	113 ± 29	.684
U‐ENaC_γ_/min (ng/minute)	0.74 ± 0.29	0.73 ± 0.28	.429
U‐ENaC_γ_/creatinine (ng/mmol)	73 ± 21	71 ± 16	.564
U‐NCC/min (ng/min)	0.58 ± 0.21	0.63 ± 0.14	.104
U‐NCC/creatinine (ng/mmol)	58 ± 15	64 ± 16	.129
U‐NKCC2/min (ng/min)	0.86 ± 0.29	0.90 ± 0.25	.402
U‐NKCC2/creatinine (ng/mmol)	88 ± 28	90 ± 25	.319

Note

Urine output, free water clearance (C_H2O_), urine excretion of sodium (U‐Na) fractional excretion of sodium (FE_Na_) creatinine clearance, urinary excretions rates of albumin (UAER), aquaporin‐2 (u‐AQP‐2/min), γ‐fraction of the epithelial sodium channel (u‐ENaC_γ_/min), sodium chloride cotransporter (u‐NCC/min), and sodium potassium chloride cotransporter (u‐NKCC2/min) and in relation to creatinine (u‐AQP‐2/creatinine, u‐ENaC_γ_/creatinine, u‐NCC/creatinine, u‐NKCC2/creatinine. Urine collected from 07.00 a.m. the day before the examination day to 07.00 a.m. on the examination day. Data are shown as means ± *SD*. Statistics are performed with paired *t* test or Wilcoxon signed‐rank test.

**TABLE 3:** Effect of furosemide on GFR and tubular function

	Baseline	0–30 min	30–60 min	60–90 min	90–120 min	*p* (GLM within)
GFR (^51^Cr‐EDTA clearance)						
Placebo	97 ± 10	96 ± 11	99 ± 11	97 ± 15	100 ± 12	<.001
Furosemide	96 ± 10	92 ± 12*	87 ± 10*	82 ± 11*	86 ± 12*	
*p (GLM between)*	.815					
Urine output (mL/min)						
Placebo	7.3 ± 1.4	4.1 ± 1.1*	6.5 ± 1.3	4.7 ± 1.8*	7.3 ± 1.8	<.001
Furosemide	7.2 ± 1.3	24.3 ± 4.4*	20.8 ± 4.0*	12.2 ± 3.6*	8.0 ± 2.8	
*p (GLM between)*	<.001					
C_H2O_ (ml/min)						
Placebo	5.0 ± 1.1	1.3 ± 1.2*	3.6 ± 1.2*	2.1 ± 1.4*	3.9 ± 1.4*	<.001
Furosemide	5.0 ± 1.3	3.6 ± 1.8*	3.2 ± 1.3*	1.2 ± 1.2*	1.3 ± 1.4*	
*p (GLM between)*	.164					
U‐Na (mmol/l)						
Placebo	25 ± 10	48 ± 20*	33 ± 13*	44 ± 13*	37 ± 10*	<.001
Furosemide	23 ± 6	109 ± 7*	107 ± 6*	108 ± 11*	91 ± 20*	
*p (GLM between)*	<.001					
FE_Na_ (%)						
Placebo	0.92 ± 0.44	1.00 ± 0.43*	1.10 ± 0.42*	1.09 ± 0.42*	1.21 ± 0.39*	<.001
Furosemide	0.85 ± 0.32	13.08 ± 2.11*	12.43 ± 1.78*	7.27 ± 1.51*	3.93 ± 1.38*	
*p (GLM between)*	<.001					

Note

Glomerular filtration rate (GFR), urine output, free water clearance (C_H2O_) and fractional excretion of sodium (FE_Na_). Urine was collected every 30 min. Data from three baseline periods are pooled and shown as one period. Data are presented as means ± *SD*. Statistics are performed with a general linear model (GLM) or paired *t* test. Statistically significant difference from baseline: * = *p* < .05.

**TABLE 4:** Effect of furosemide on excretion of proteins from aquaporin‐2 channels and tubular channels that mediate sodium transport

	Baseline	0–30 min	30–60 min	60–90 min	90–120 min	*p* (GLM within)
U‐AQP2 (ng/minute)						
Placebo	1.19 (0.97;1.41)	1.20 (1.03;1.39)	1.19 (1.01;1.39)	1.09 (0.95;1.27)*	1.31 (1.03;1.43)	<.001
Furosemide	1.21 (1.04;1.39)	3.42 (2.83;3.81)*	2.78 (2.18;3.81)*	1.95 (1.55;2.76)*	1.68 (1.30;2.01)*	
*p (GLM between)*	<.001					
U‐AQP2/creatinine (ng/mmol)						
Placebo	115 (104;136)	211 (153;249)*	134 (111;155)	179 (140;132)*	118 (105;143)	.001
Furosemide	123 (101;135)	105 (85;129)	100 (73;145)	112 (83;179)	139 (91;205)	
*p (GLM between)*	.059					
U‐ENaC_γ_ (ng/minute)						
Placebo	0.66 (0.52;0.80)	0.63 (0.45;0.83)	0.57 (0.45;0.80)	0.59 (0.42;0.66)	0.65 (0.44;0.77)	.017
Furosemide	0.57 (0.48;0.84)	0.76 (0.53;0.89)	0.55 (0.45;0.81)	0.74 (0.62;0.84)*	0.77 (0.64;0.94)*	
*p (GLM between)*	.355					
U‐ENaC_γ_/creatinine (ng/mmol)						
Placebo	61 (54;70)	105 (87;139)*	63 (53;83)	80 (66;116)*	57 (52;80)	<.001
Furosemide	64 (56;71)	19 (14;28)*	18 (15;29)*	47 (37;60)*	64 (55;101)	
*p (GLM between)*	<.001					
U‐NCC (ng/min)						
Placebo	0.58 (0.51;0.77)	0.58 (0.46;0.65)*	0.58 (0.53;0.64)	0.53 (0.44;0.66)	0.59 (0.50;0.67)	<.001
Furosemide	0.57 (0.52;0.71)	1.53 (1.35;1.97)*	1.36 (1.24;1.67)*	1.02 (0.83;1.23)*	0.69 (0.65;0.93)*	
*p (GLM between)*	<.001					
U‐NCC/creatinine (ng/mmol)						
Placebo	65 (57;74)	91 (72;112)*	69 (56;79)	82 (61;103)*	57 (47;73)	.003
Furosemide	60 (49;68)	54 (37;68)*	46 (38;65)	69 (46;84)	71 (54;95)	
*p (GLM between)*	.007					
U‐NKCC2 (ng/min)						
Placebo	0.96 (0.78;1.14)	0.94 (0.75;1.03)*	0.93 (0.78;1.07)	0.87 (0.69;1.03)*	0.97 (0.70;1.17)	<.001
Furosemide	1.00 (0.75;1.11)	4.50 (3.73;5.02)*	3.51 (2.87;4.23)*	2.41 (2.03;2.59)*	1.65 (1.39;2.28)*	
*p (GLM between)*	<.001					
U‐NKCC2/creatinine (ng/mmol)						
Placebo	84 (75;118)	160 (106;197)*	111 (82;118)	122 (92;156)*	92 (68;112)*	<.001
Furosemide	91 (69;104)	111 (93;169)*	106 (96;157)*	155 (124;179)*	155 (132;197)*	
*p (GLM between)*	.245					

Note

Aquaporin‐2 (u‐AQP‐2/min), γ‐fraction of the epithelial sodium channel (u‐ENaC_γ_/min), sodium chloride cotransporter (u‐NCC/min), and sodium potassium chloride cotransporter (u‐NKCC2/min) and in relation to creatinine (u‐AQP‐2/creatinine, u‐ENaC_γ_/creatinine, u‐NCC/creatinine, u‐NKCC2/creatinine. Urine was collected every 30 min. Data from three baseline periods are pooled and shown as one period. Data are shown as medians with 25 and 75 percentiles in brackets *p*‐value represents the probability of difference in response to hypertonic saline (response from baseline to hypertonic saline) between treatments Statistics are performed with a general linear model (GLM), or Wilcoxon signed‐rank test. Data were logarithmic transformed before GLM was performed. Statistically significant difference from baseline: * = *p* < .05.

### 3.5 GFR and tubular function after furosemide

Table 3 shows the effect furosemide on GFR, urine output (UO), C_H2O_, and FE_Na_. Urine output and FE_Na_ increased markedly after furosemide while GFR decreased. Total diuresis was 1959 ml after furosemide and 678 ml after placebo with a total difference of 1,281 ml (*p* < .001). C_H2O_ decreased after both placebo and furosemide but C_H2O_ had a different response pattern after placebo and furosemide. The initial decrease in C_H2O_ after placebo was attenuated after furosemide, but was exaggerated in the last two clearance periods.

### 3.6 Urinary excretion of proteins from ENaC_γ_, AQP2, NCC, and NKCC2

Table 4 shows the furosemide‐induced changes in u‐AQP2, u‐ENaC_γ_, u‐NCC, and u‐NKCC2. Different responses in u‐AQP2, u‐ENaC_γ_, u‐NCC, and u‐NKCC2 were observed after placebo and furosemide.

U‐AQP2, u‐ENaC_γ_, u‐NCC, and u‐NKCC2 were mainly unchanged after placebo. Small differences were seen in one or two clearance periods with‐out a clear pattern for any of the proteins. The only exception is the creatinine adjusted u‐NKCC2 excretion where three of four clearance periods show a higher excretion level.

After furosemide, u‐AQP2 excretion rate increased, but this increase was not present when u‐AQP2 was adjusted for creatinine. U‐ENaC_γ_ decreased after furosemide when adjusted for creatinine. Time adjusted u‐ENaC_γ_ increased slightly. U‐NCC excretion increased after furosemide when time‐adjusted but when adjusted for creatinine NCC excretion was mainly unchanged.

Similarly, when time‐adjusted U‐NKCC2 increased markedly in the first clearance period after furosemide and decreased toward baseline in the following clearance periods. Creatinine adjusted u‐NKCC2 increased steadily throughout the clearance periods.

### 3.7 Vasoactive hormones in plasma

Plasma‐AVP, PRC, p‐Ang II, and p‐Aldo were not different at baseline (Table 5). P‐AVP was not changed by furosemide. Furosemide significantly increased PRC, p‐Ang II, and p‐Aldo.

**TABLE 5:** Effect of furosemide on vasoactive hormones

	Baseline	1 hr after intervention (60 min)	2 hr after intervention (120 min)	*p*‐value (difference in response)
p‐AVP (ng/L)				
Placebo	0.20 (0.20;0.40)	0.20 (0.10;0.30)	0.30 (0.20;0.35)	.317
Furosemide	0.30 (0.20;0.40)	0.30(0.20;0.45)	0.30(0.20;0.45)	
PRC (ng/L)				
Placebo	6.9 (5.3;9.5)	5.8 (3.9;8.1)	5.4 (4.2;7.3)*	.001
Furosemide	6.8 (5.1;10.2)	15.8 (11.9;26.9)*	15.4 (10.7;25.0)*	
p‐AngII (ng/L)				
Placebo	10 (7;14)	10 (7;14)	9 (7;14)	<.001
Furosemide	10 (7;14)	22 (18;37)*	24 (15;31)*	
p‐Aldo (pmol/L)				
Placebo	106 (59;148)	74 (48;134)*	77 (52;107)*	<.001
Furosemide	71 (49;123)	354 (146;469)*	224 (159;454)*	

Note

Plasma concentrations arginine vasopressin (p‐AVP), renin (PRC), angiotensin II (p‐AngII), and aldosterone (p‐Aldo) were measured before and 60 and 120 min after injection of either placebo or furosemide. Data are shown as medians with 25 and 75 percentiles in brackets. *p*‐value represents the probability of difference in response to furosemide (response from baseline to 60 min) between treatments. Students *t* test was used to test the difference in response to furosemide between treatments. Wilcoxon signed‐rank test was used to test statistically significant difference from baseline, * = *p* < .05.

### 3.8 Blood pressure (BP)

Hemodynamic variables are shown in Table 6. Systolic BP (SBP) was not altered by furosemide but diastolic BP (DBP) and HR increased after furosemide.

**TABLE 6:** Effect of furosemide on hemodynamic variables

	Baseline	0–30 min	30–60 min	60–90 min	90–120 min	*p* (GLM within)
SBP (mmHg)						
Placebo	119 ± 9	119 ± 11	120 ± 10	120 ± 9	120 ± 9	.105
Furosemide	118 ± 9	120 ± 9	119 ± 9	119 ± 10	119 ± 10	
*p (GLM between)*	.863					
DBP (mmHg)						
Placebo	63 ± 6	63 ± 5	63 ± 5	63 ± 6	63 ± 6	<.001
Furosemide	61 ± 6	67 ± 5*	68 ± 6*	68 ± 7*	68 ± 6*	
*p (GLM between)*	.036					
HR (beats/min)						
Placebo	58 ± 8	58 ± 8	58 ± 8	59 ± 8*	60 ± 9*	<.001
Furosemide	57 ± 9	56 ± 10	60 ± 10*	64 ± 11*	65 ± 10*	
*p (GLM between)*	.184					

Note

Systolic and diastolic blood pressure (SBP, DBP) and heart rate (HR) were measured every 30 min. Data from three baseline measurements are pooled and shown as one period. Data are presented as means ± *SD*. Statistics are performed with a general linear model (GLM) or paired *t* test. Statistically significant difference from baseline: * = *p* < .05.

### 3.9 Correlations

Correlation between changes in ECW and ICW and changes in bodyweight, vasoactive hormones, urinary flow, and proteins that mediate tubular sodium and water transport after infusion of furosemide were examined. The biggest response to furosemide was observed in the 1‐hr period after furosemide was given and therefore the changes in this period were evaluated. Multivariate analyses were not performed due to patient number.

Correlations for the change in ECW are shown in Table 7. The changes in ECW correlated well with change in bodyweight, urine output, and u‐NKCC2. The change in ICW had a poor correlation with all variables with correlation coefficients between −0.3 and 0.3 (*p* = NS for all).

**TABLE 7:** Correlations between change in ECW and change in bodyweight, urinary output, vasoactive hormones, and proteins of tubular sodium and aquaporin channels after infusion of furosemide

		Correlation (*r*)	*p*
ECW	Bodyweight	0.752	<.001
	Urine output	−0.739	<.001
	U‐NKCC2/min	−0.670	.002
	U‐NKCC2/creatinine	−0.595	.007
	U‐NCC/min	0.501	.029
	U.NCC/creatinine	0.092	NS
	U‐ENaC_γ_/min	0.490	.033
	U‐ENaC_γ_/creatinine	0.348	NS
	U‐AQP2/min	−0.156	NS
	U‐AQP2/creatinine	0.584	.009
	PRC	−0.160	NS
	P‐Aldo	−0.225	NS
	P‐AngII	0.125	NS
	P‐AVP	−0.272	NS

Note

Aquaporin‐2 (u‐AQP‐2/min), γ‐fraction of the epithelial sodium channel (u‐ENaC_γ_/min), sodium chloride cotransporter (u‐NCC/min) and sodium potassium chloride cotransporter (u‐NKCC2/min) and in relation to creatinine (u‐AQP‐2/creatinine, u‐ENaC_γ_/creatinine, u‐NCC/creatinine, u‐NKCC2/creatinine. Plasma concentrations arginine vasopressin (p‐AVP), renin (PRC), angiotensin II (p‐AngII), and aldosterone (p‐Aldo). Correlation was performed with Pearson's correlation.

**FIGURE 3:**
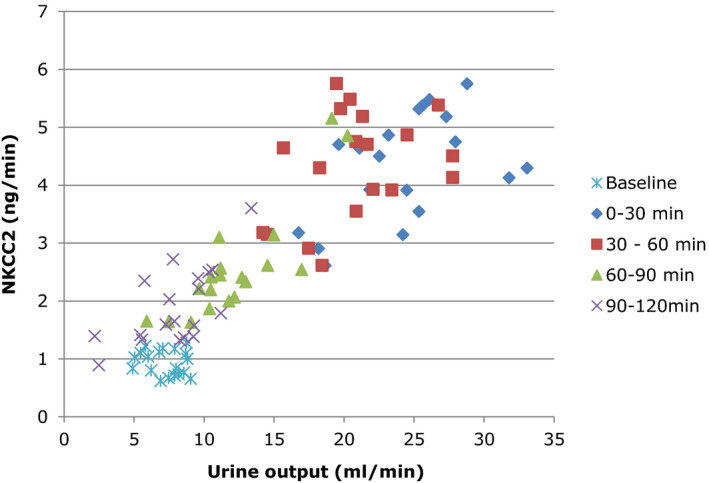
Urine output in relation to urinary u‐NKCC2 excretion rate for all clearance periods

The relation between absolute values for urine output and u‐NKCC2 for all clearance periods is shown in Figure 3. There was a significant correlation between urine output and u‐NKCC2 for all periods except for the baseline period (baseline: *r* = −0.166, *p* = .497; 0–30 min: *r* = 0.498, *p* = .030, 30–60 min; *r* = 0.674, *p* = .002; 60–90 min: 0.849; *p* < .001; 90–120 min: 0.649, *p* = .003; All periods combined; *r* = 0.882, *p* < .001).

## 4 DISCUSSION

In this study, we estimated BIS during an acute intervention with furosemide. We observed an expected increased diuresis with a subsequent reduction in bodyweight after furosemide. We found a positive correlation between the decrease in ECW and a decrease in bodyweight and a negative correlation between the decrease in ECW and the increase in urinary output. This suggests that BIS can detect acute changes in body fluid and sodium changes. In addition, the change in u‐NKCC2 after furosemide correlated with the change in urine output and change in u‐NKCC2.

In this study, the mean reduction in bodyweight after furosemide was 1.5 kg equal to 1.5 L of fluid. As expected ECW decreased and the average decrease was 1.2. This confirms the previous finding that estimation of ECW using BIS can be used to detect volume depletion after furosemide. Previous studies were performed in patients with overhydration caused by liver, heart, and kidney disease and the new finding in this study is that the same finding is present in healthy normohydrated subjects (Nagayama et al., 2019; Ng Kam Chuen et al., 2009; Ohara et al., 2019).

Surprisingly the ICW increased 0.5 L. ECW decreased 1.1 L and this sums to a total decrease of 0.7 L estimated by BIS which is 0.8 L less than the decrease in total bodyweight. An increase in ICW is not observed in previous studies using furosemide (Nagayama et al., 2019; Ng Kam Chuen et al., 2009; Ohara et al., 2019). The explanation for the discrepancy found in this study is not clear. Several issues could be involved.

Our subjects arrived fasting for the examinations and were given 1925 ml of tap water during the entire examination and 700 ml tap water was given after furosemide or placebo was injected. After placebo, diuresis was almost equal to the oral tap water given, but after furosemide diuresis was 1.3 liters higher than the tap water given. Whereas tap water contains a minimal amount of solutes, furosemide creates an electrolyte diuresis containing both water and sodium. Sodium in urine is initially taken from plasma. In our study p‐Na decreased after furosemide and a similar decrease was observed after placebo. Hence, after furosemide additional, sodium must be added to plasma from other compartments such as skin and bone tissue. This suggests that a new balance is created which could include the addition of sodium from tissue compartments (e.g., bone and bone marrow) which may influence the reliability of BIS. This might explain the increased ICW found.

In addition, volume status may be important for the BIS method. BIS was previously used in patients with or at risk of volume overload whereas this study was performed according to OH estimation in normohydrated or slightly dehydrated subjects who are reduced in body fluid (Cichoz‐Lach & Michalak, 2017; Ersoy Dursun et al., 2019; Hur et al., 2013; Nagayama et al., 2019; Ng Kam Chuen et al., 2009; Ohara et al., 2019; Onofriescu et al., 2014). BIS may be less reliable in estimating ICW in a setting of normohydration and a reduction in body fluid from the normohydration state, which might be the explanation for our finding.

The acute intervention and relative short follow‐up may compromise the method. In patients with liver cirrhosis or chronic kidney injury, several studies have tried to estimate fluid status volume overload and, for example, the need for ultrafiltration in hemodialysis patients and follow‐up measurement were performed with a daily to weekly interval (Cichoz‐Lach & Michalak, 2017; Ersoy Dursun et al., 2019; Hur et al., 2013; Nagayama et al., 2019; Ng Kam Chuen et al., 2009; Ohara et al., 2019; Onofriescu et al., 2014). A similar approach, with daily BIS measurements being performed in critically ill patients in intensive care units, BIS has been used to estimate and monitor fluid status (Fülöp et al., 2017; Malbrain et al., 2014). Follow‐up measurement in this study were performed within 1 and 2 hr and the BIS method may need a longer period for the body to reach the steady state to give an accurate estimate of ECW and ICW and subsequent TBW, and we cannot exclude that the measurements were close to the intervention made and we would get different results regarding ICW with a longer observation period. Further studies are needed to examine if our finding is a true change in ICW or a matter of short‐comings of the BIS method.

Furosemide inhibits the cotransporter NKCC2 in the ascending limp of the loop of Henle (Huang et al., 2017). As expected, we observed that tubular sodium excretion (Fe_Na_) increased after furosemide. The novel finding in this study is that u‐NKCC2 also increases after furosemide. Intuitively one would think that inhibition of a channel or cotransporter would lead to a decrease in the amount of urinary protein material measured, but in this study the opposite—an increased excretion—is observed. There was a significant positive correlation between urinary NKCC2 concentration and urine output in the different collection periods. In addition, the changes in u‐NKCC2 excretion after furosemide correlated well with changes in ECW. The explanation for the increased u‐NKCC2 excretion after furosemide is not certain. Since u‐NKCC2 concentration is unchanged after furosemide it may just be a shedding of proteins from the apical membrane due to an increased flow in the urinary space in the thick ascending limb. In addition, apical expression of NKCC2 may have changed. The binding of furosemide can affect endocytosis which could increase the delivery of protein material from NKCC2 to the urine (Bahro et al., 1988). The apical expression could also be linked to other factors than furosemide demonstrated in rodents where expression was modulated by salt intake (Haque et al., 2011). Intracellular trafficking of NKCC2 is affected by several hormones including AVP, parathyroid hormone (PTH), atrial natriuretic peptide (ANP) and glucagon and local factors including nitric oxide, endothelin‐1, and norepinephrine (Ares et al., 2011). AVP increases exocytosis and activity of NKCC2 via a cAMP‐dependent pathway (Ares et al., 2011). In this study p‐AVP is unchanged after furosemide, and AVP is therefore not the likely explanation for increased NKCC2 excretion observed in this study. The involvement of the other hormones and local factors are not evaluated in this study. To date, three different NKCC2 isoforms derived from differential splicing are known: NKCC2A, NKCC2B, and NKCC2F, but the functional significance of the three isoforms is uncertain (Ares et al., 2011; Oppermann et al., 2007; Schieβl et al., 2013). The radioimmunoassay method used to determine NKCC2 levels in this study cannot distinguish between the three isoforms, but a method that can distinguish between the isoforms could help us to estimate the significance of the three NKCC2 isoforms and help to determine if the increased urinary NKCC2 during furosemide is a shedding phenomenon or connected to other mechanisms. The main issue that remains to be resolved is if the u‐NKCC2 after furosemide can be used as a measure of transport activity, and further studies are needed to resolve this issue and if u‐NKCC2 measurement has clinical relevance.

U‐ENaC_γ,_ u‐NCC, and u‐AQP2 excretion rates (ng/minute) all increased after furosemide. The increase in U‐ENaC_γ_ and u‐AQP2 excretion rates (ng/minute) is in accordance with the previous finding from our laboratory (Mose et al., 2019; Starklint et al., 2005). The increase in u‐NCC excretion rate is to our knowledge a novel finding. As discussed for u‐NKCC2 it is not known if u‐NCC protein excretion reflects activity. If it reflects activity the increased urinary protein material could indicate a compensatory increase in cotransporter activity. This in mind urinary flow is increased after furosemide which may increase shedding of protein material from tubular epithelial cells. As for NKCC2, the clinical use of these findings still needs to be established.

## 5 CONCLUSION

We found that BIS can detect acute changes in body water content but the method may be limited to estimation of ECW. BIS demonstrated that furosemide increases ICW which might be explained by an extracellular sodium loss. And finally urinary proteins from NKCC2 increase after furosemide with a good correlation with diuresis end the decrease in ECW.

## DISCLOSURES

All authors declare no conflict of interests.

## AUTHORS' CONTRIBUTIONS

All authors have consented and contributed to the publication. AE Oczachowska‐Kulik and JN Bech designed the project. AE Oczachowska‐Kulik, JN Bech, and RA Fenton performed the experiments and laboratory analysis, M Frank Holden performed statistical analysis, and M Frank Holden, AE Oczachowska‐Kulik, JN Bech, and RA Fenton wrote and edited the manuscript.
